# Different modalities of measuring life engagement in people living with schizophrenia spectrum disorders: A preliminary analysis

**DOI:** 10.1192/j.eurpsy.2024.454

**Published:** 2024-08-27

**Authors:** C. Cerati, I. Calzavara-Pinton, G. Nibbio, A. Cicale, D. Zardini, M. Italia, L. Altieri, J. Lisoni, G. Deste, S. Barlati, A. Vita

**Affiliations:** ^1^Department of Biomedical Sciences and Translational Medicine, University of Brescia; ^2^Department of Mental Health and Addictions, ASST Spedali Civili di Brescia, Brescia, Italy

## Abstract

**Introduction:**

The concept of “life engagement” encompasses several aspects of one’s life, including personal well-being, contentment, purpose, and engagement in meaningful activities. In 2006, the group led by Scheier designed a 6-item scale to measure this concept in the general population: the Life Engagement Test (LET), however, this tool was never validated in clinical populations (Scheier *et al.* 2006 J Clin Psychiatry 2006; 29 291-298). In subjects living with schizophrenia life engagement can be measured through the Positive and Negative Syndrome Scale-Life Engagement (PANSS-LE), derived by isolating 11 items (i.e., N01, N02, N03, N04, N05, N06, G06, G07, G13, G15, G16) from the PANSS (Correll *et al.* 2022 J Clin Psychiatry 2022; 83-4) (Correll *et al.* 2022 J Clin Psychiatry 2022; 83-5).

**Objectives:**

The aim of this study was to investigate the clinical and functional correlates of two different measures of life engagement in a cohort of individuals living with schizophrenia spectrum disorders (SSD).

**Methods:**

Ninety-five subjects living with SSD recruited from the ASST Spedali Civili of Brescia (Italy) were included in the preliminary ad-interim analysis of the present study: for each patient information regarding the clinical presentation were measured with the Clinical Global Impression (CGI) scale, the Health of the Nation Outcome Scales (HoNOS), the Brief Negative Symptoms Scale (BNSS) and the PANSS; additionally, information related to the psychosocial functioning were collected through the Global Assessment of Functioning (GAF) scale; finally, life engagement was evaluated through the LET and the PANSS-LE. Spearman’s correlations were performed using SPSS v28 and p values < 0.05 were considered significant.

**Results:**

Both the LET and the PANSS-LE were correlated with the CGI (p=0.002 and p<0.001 respectively), but only the PANSS-LE was found to be correlated with the GAF (p<0.001), the BNSS (p<0.001) and the HoNOS (p<0.001).

**Conclusions:**

The concept of life engagement is of growing interest for healthcare professionals working in the mental health field, in line with the concept of reaching a full functional recovery and considering patient-reported outcomes. From our study it is evident that life engagement in individuals living with SSD is better characterized through the PANSS-LE rather than the LET, as the former is more specific to define the complexity of the SSD symptomatology.

**Disclosure of Interest:**

None Declared

